# Best practices and challenges in executing large scale pilots for eHealth deployment and managing innovation: the GATEKEEPER experience

**DOI:** 10.3389/fdgth.2026.1730232

**Published:** 2026-05-29

**Authors:** Davide Piaggio, Jordi de Batlle, Alba M. Gallego Montejo, Gloria Cea Sánchez, Pedro Checa Rifá, Alessia Maccaro, Rosana Angles Barbastro, Maria Teresa Hurtado, Jon Eneko Idoyaga Uribarrena, Irati Erreguerena Redondo, Maria Krini, Maria Matsangidou, Ioanna Drympeta, Konstantinos Votis, Alessio Antonini, Przemyslaw Kardas, Pawel Lewek, Julia Schellong, Francesco Giuliani, Franco Mercalli, Giuseppe Fico, Leandro Pecchia, the GATEKEEPER Consortium

**Affiliations:** 1 Applied Biomedical Signal Processing Intelligent eHealth Lab, University of Warwick, Coventry, United Kingdom; 2 European Alliance of Medical and Biological Engineering and Science (EAMBES), Leuven, Belgium; 3 Group of Translational Research in Respiratory Medicine, Institut de Recerca Biomèdica de Lleida – Fundació Dr. Pifarré, IRBLleida, Lleida, Spain; 4 Centro de Investigación Biomédica en Red de Enfermedades Respiratorias (CIBERES), Madrid, Spain; 5 Life Supporting Technologies-LifeSTech, Universidad Politécnica de Madrid, Madrid, Spain; 6 Unidad de Innovación, Hospital de Barbastro, Servicio Aragonés de Salud, Barbastro, Spain; 7 Internationalization and Project Coordination Unit, Biobizkaia Health Research Institute, Barakaldo (Bizkaia), Spain; 8 Biosistemak Institute for Health Systems Research, Bilbao, Spain; 9 Network for Research on Chronicity, Primary Care, and Health Promotion (RICAPPS), Bilbao, Spain; 10 Cyprus Association of Cancer Patients and Friends (PASYKAF), Nicosia, Cyprus; 11 CYENS Centre of Excellence, Nicosia, Cyprus; 12 Information Technologies Institute, Centre for Research and Technology, Thessaloniki, Greece; 13 Knowledge Media Institute, The Open University, Milton Keynes, United Kingdom; 14 Department of Family Medicine, Medical University of Lodz, Łódz, Poland; 15 Faculty of Medicine Carl Gustav Carus, Technical University Dresden, Germany; 16 Innovation and Research Unit, Casa Sollievo della Sofferenza Research Hospital, San Giovanni Rotondo, Italy; 17 MultiMed Engineers Srls, Parma, Italy; 18 Department of Engineering, University Campus Bio-Medico, Rome, Italy

**Keywords:** best practices, business intelligence, digital health, eHealth, GATEKEEPER EU, large scale pilot, lessons learned, project management

## Abstract

The large-scale deployment of digital health solutions requires robust operational frameworks capable of coordinating heterogeneous settings, diverse stakeholders, and complex technical infrastructures. However, actionable guidance for executing federated, multinational eHealth pilots remains limited in the implementation literature. Methods: Using a mixed-methods approach, including iterative focus groups, co-creation sessions, and a Delphi study, we developed and refined a practice-derived management framework over four years within the GATEKEEPER project (EU Horizon 2020, Grant Agreement No. 857223). The study involved a federation of four European large-scale pilots. A panel of 23 experts ranked 45 best practices across six operational domains: Engagement, Intervention, Monitoring and Control, Planning, Recruitment, and Other. Results: The resulting framework integrates a structured definition of operative key performance indicators, standardised reporting and analysis tools, and a Business Intelligence dashboard to support real-time monitoring and decision-making across the preparation, deployment, and running phases of large-scale pilots. Among the ranked best practices, usability testing, user-centred digital tool design, and active recruitment strategies emerged as top priorities across pilot sites. Discussion: This management framework addresses a critical gap in implementation science by offering actionable, consensus-validated guidance for coordinating large, distributed, multi-site digital health deployments. The GATEKEEPER experience demonstrates how structured operational governance and shared performance monitoring can support the execution of complex eHealth pilots, with insights that may inform future large-scale initiatives seeking sustainable and patient-centred digital health integration.

## 1 Introduction

Healthcare 4.0 represents a breakthrough in the way healthcare services are delivered and managed, leveraging the power of digital technologies and data-driven approaches to enhance healthcare outcomes (1). Building on top of this concept, Healthcare 5.0 strives for an increased patient-centred and personalised care approach. The concept of eHealth encompasses the integration of various technological domains, including the Internet of Things (IoT), artificial intelligence (AI), big data analytics, and computing, enabling the seamless exchange of medical information, enhancing clinical decision-making, and facilitating remote patient monitoring (46). The development of these technologies decreases healthcare costs and non-necessary hospitalisation (2, 3), facilitates the remote access to the healthcare system (4, 5), improves the capabilities of care delivery (4), enhances the quality of life (4), and optimises the efficiency of care (6). Therefore, eHealth implementation is a need and a challenge that many healthcare systems are facing or will face in the near future (7).

In order to live up to the expectations raised by the results of small-scale Pilots, the implementation of large-scale Pilots (LSPs) becomes fundamental in the deployment path of eHealth (8). Nevertheless, the large-scale implementation of eHealth introduces many challenges and considerations in the fields of execution, ethics and legal aspects, data protection and management, and technical development (9). As healthcare systems become increasingly interconnected and reliant on digital technologies, safeguarding patient information and ensuring the seamless exchange of data between different platforms and providers become critical areas of focus (2, 6). Moreover, other challenges can be related to the implementation costs (2, 4, 10), the lack of management support and poor leadership, inadequate planning, organisational structure, as well as poor communication and engagement (11), or the use of AI itself (12).

The European Commission has invested efforts to innovate and implement eHealth through its Horizon 2020 and Horizon Europe research and innovation programs. Among those, the largest funds have been allocated to LSPs and platform projects, which have been further aided by initiatives such as OPEN DEI (https://www.opendei.eu), supporting the implementation of EU digitisation policies, focusing on LSPs and platform projects within the healthcare domain. The GATEKEEPER project (https://www.gatekeeper-project.eu; https://cordis.europa.eu/project/id/857223), aiming to foster collaboration among healthcare providers, businesses, entrepreneurs, elderly citizens, and their communities to promote healthier and independent living for aging populations, stands as the largest of the EU-funded LSPs. GATEKEEPER conducted up to 30 federated implementation trials addressing nine Reference Use Cases (RUCs) in seven European [Aragon, Basque Country, Cyprus, Greece, Poland, Puglia, Saxony, and the UK (Milton Keynes and Bangor)] and three Asian (Singapore, Hong Kong, and Taiwan) pilots, involving tens of thousands of participants (13). Each of GATEKEEPER's implementation sites mobilized collaborations among healthcare organisations, industry partners, academia, and government; with each GATEKEEPER's RUCs focusing on aspects such as lifestyle interventions, chronic disease management (e.g., chronic obstructive pulmonary disease, heart failure, diabetes and hypertension), Parkinson's disease support, stroke prevention, multimorbid patient care, cancer management, and COVID-19 response (13). The GATEKEEPER project focus is on interconnected technologies to improve patients’ and citizens’ quality of life, for example through the use of wearables, digital tools, AI-driven solutions, etc. all connected to a dedicated platform. Further details can be read in these publications (13, 14).

Several well-established frameworks exist to guide the implementation and evaluation of eHealth technologies. The NASSS framework (15) addresses factors influencing technology adoption and sustainability, while the Consolidated Framework for Implementation Research (CFIR) (16) provides a taxonomy of constructs influencing implementation success. Similarly, the RE-AIM framework (17) offers a structure for evaluating interventions across reach and maintenance dimensions. While these frameworks are invaluable for understanding what factors influence success and how to evaluate outcomes, they do not provide operational guidance for the day-to-day coordination challenges inherent in executing large-scale, multi-site eHealth pilots. The management of LSPs presents unique challenges that extend beyond implementation determinants. These include coordinating federated pilot sites with heterogeneous settings and stakeholder groups, establishing efficient reporting mechanisms, and fostering collaboration across distributed teams. As members of the GATEKEEPER LSP management team, the authors identified a significant gap in the literature regarding these execution-phase hurdles.

Consequently, this manuscript aims to identify the primary operational challenges and propose a management framework derived from the direct experience acquired during the GATEKEEPER project. These findings are further bolstered by a Delphi study involving experts from other LSPs under the OPEN DEI umbrella. This paper provides a practice-derived framework that complements existing implementation and evaluation models by offering concrete tools and consensus-based best practices. Notably, this work is complementing a publication by the same team, which analysed and presented a framework for the ethical challenges faced by LSP projects (14), thereby providing a comprehensive overview of both the operational and moral complexities of large-scale eHealth deployment.

## 2 Methods

The applied methodology involved a triangulation of qualitative and quantitative methods. Qualitative methods, in fact, can be complementary to quantitative ones, when used as a crucial preliminary step, and provide Supplementary Data for further validation (18). In our specific case, these methods were applied iteratively over a four-year period as part of a longitudinal, participatory research process. Rather than being conducted as isolated or countable events, qualitative activities evolved over time and often overlapped in terms of participants and objectives. Specifically, the following approaches were employed:

1. Focus groups, co-creation sessions, and learning by doing, used for developing and refining our proposed framework as well as for extracting the best practices for LSP management.
2. Internal validation of both our framework and best practices through focus groups and co-creation sessions based on the GATEKEEPER LSP experience.
3. Multi-project expert consensus building on best practices via a Delphi study with experts from the OPEN DEI cluster.

**Figure 1:**
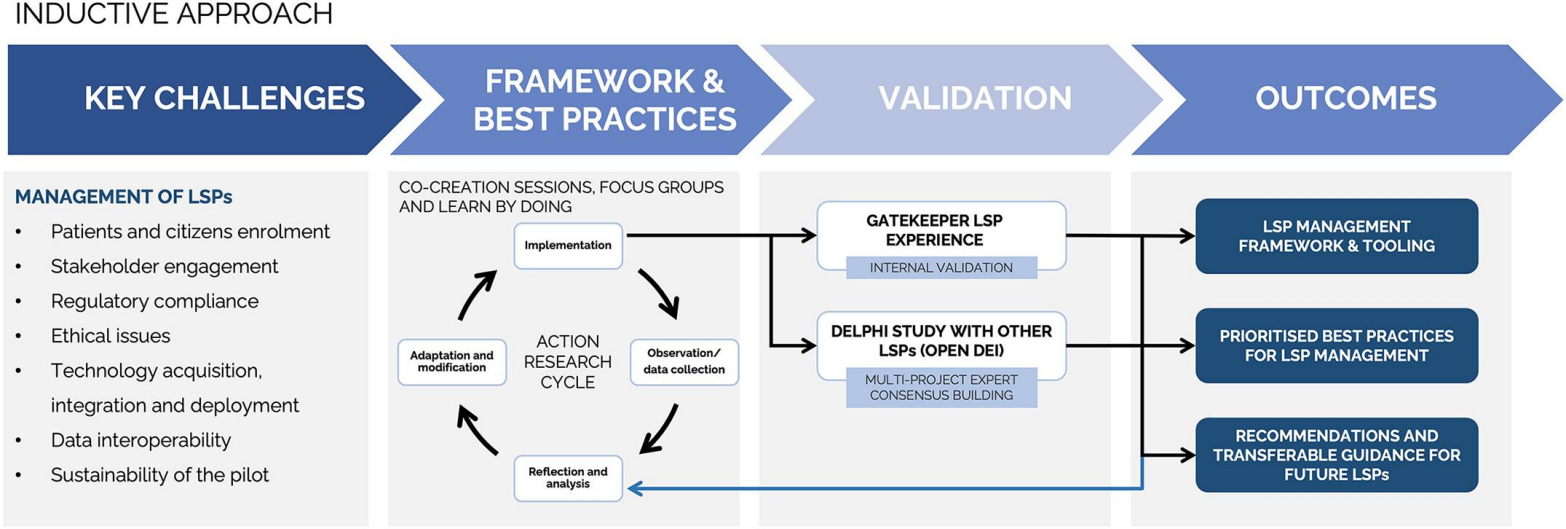
Methods applied throughout the project. The figure presents the key challenges, the development and validation process of the LSP management framework, and the main outcomes.

In general, the overarching applied method followed an inductive approach (Figure 1), which allows the formulation of theories starting from several observations (19). In particular for the assessment of best practices, this method initiates with the analysis of key challenges in LSP management within the context of eHealth deployment. Leveraging collaborative engagement techniques and an iterative development process, the framework is progressively refined through the insights and experiences of key LSPs. The following sections give further details on our methodologies.

### 2.1 Framework definition and refinement

This section outlines the methodology employed to establish, refine and iteratively optimise the management framework and the tools that have been applied for an effective monitoring, evaluation, and reporting of technical issues during the execution of the GATEKEEPER LSP.

**Figure 2:**
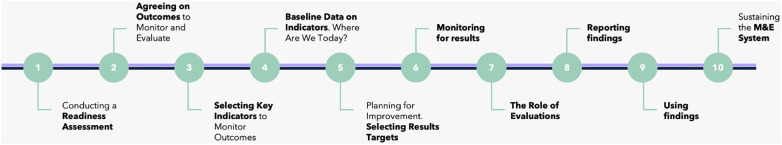
Ten steps model adopted for designing and building the GATEKEEPER management framework ^17^. The methodology outlines the ten steps followed to define the KPIs and tools for monitoring and evaluating LSP performance.

Based on the model proposed in “Ten Steps to a Results-Based Monitoring and Evaluation System” (20), as illustrated in Figure 2, the first step in defining this framework was to establish the specific objectives and results for each of the stages of the Pilot execution—preparation, deployment and running—and to translate them into Operative Key Performance Indicators (KPIs). Operative KPIs allow to measure the implementation and performance of the Pilot, identify areas for improvement, and facilitate the decision-making process. Subsequently, the description, the level of detail and the required complementary information (such as the type of intervention and the category of user involved) for each KPI should be defined. The next step involved the selection, definition, and creation of the tool for periodically reporting this information, i.e., the reporting tool. It is important to consider that this tool should be accessible, shareable, user-friendly, and adaptable to each specific Pilot characteristic while maintaining the standardised structure to facilitate the extraction, processing, and analysis of the data. Once the reporting tool—*Operative KPIs* in a MS Excel spreadsheet (see Supplementary Data 1)*—*was defined, prepared and ready for use, the next step entailed sharing and validating it with Pilots. Following the suggestions from the Pilots, some operative KPIs descriptions included were revised, thereby concluding the definition and validation phase of the reporting tool.

While the reporting tool based on the MS Excel spreadsheet provides the capability to collect, monitor and evaluate the progress of a small Pilot, this approach reduces its efficiency when applied to an LSP due to the large volume of data collected during its execution. In order to enhance the monitoring and informed decision-making process, the integration of a second tool—a Business Intelligence dashboard—has been deemed necessary. This analytical tool (*LSP Progress execution dashboard* in MS Power BI) enables the visualisation of different metrics through interactive indicators, tables and charts, accelerating the decision-making process, and the realisation of strategic plans as described in the article authored by Sutner (21).

The next step is focused on the conceptualisation, design, and implementation of the *LSP Progress execution dashboard* in MS Power BI, following the reporting tool data format and sections and adhering to the usability guidelines (22). After the development of the tools, *Operative KPIs* in the MS Excel spreadsheet and *LSP Progress execution dashboard* in MS Power BI, establishing the frequency for reporting collection became essential. This has been initially set at once per month in GATEKEEPER. Further refinements took place, and new tools have been incorporated over the months according to the Pilot phase, following the same process, until reaching the final version of our management framework presented in the Results (section “The GATEKEEPER management framework”).

### 2.2 Ranking and consensus of best practices via a Delphi study

This section illustrates the methods applied for collecting, evaluating, and building consensus on the lessons learned, with the overall aim of finding consensus on possible best practices for the deployment of digital solutions in health in Europe and ranking them.

In order to draft a first list of best practices we performed three 2 h-workshops with co-creation sessions with the Pilot Representatives and the LSP team (total participants being 15), each followed by a debrief traditional focus group among the GATEKEEPER LSP management team. These events were always moderated by one or more members of the LSP team with previous experience in managing this kind of activities and took place in a comfortable hybrid environment with some participants present in person and others connecting remotely. The remote attendees could rely on the support of an ad-hoc LSP team resource to make sure there was coordination among the two kinds of participants. Online collaborative tools such as Miro and Vevox were used as tools during these events. Dedicated notes were taken by one or more members of the LSP team. This allowed us to draft a list of best practices, which were then independently coded by two members of the LSP team and reviewed by a third. Consequently, they were divided into six meaningful categories, namely Planning, Recruitment, LSP monitoring and control, Engagement, Intervention, and Other. The final draft of best practices and their codes were shared with the Pilot Representatives for feedback and internal validation. A questionnaire for guiding the Delphi study was then created based on the above-mentioned best practices and categories.

A Delphi study provides for an iterative multistage process through which the consensus of a group of expert panellists is reached (23). In order to minimise the “bandwagon effect”, i.e., preventing the authority or reputation of an individual from dominating others (18), it was decided to conduct a blind online Delphi survey. The target panellists were the key partners selected to run LSP projects by the European Commission, such as the GATEKEEPER project and other projects part of the Health and Care Cluster of Aligning Reference Architectures, Open Platforms and Large-Scale Pilots in Digitising European Industry (Open DEI). In particular, in GATEKEEPER all the local PIs for the Pilots were responsible of research centres with a European Innovation Partnership on Active and Healthy Ageing (EIPonAHA) score of at least 2 stars. These selected potential panellists are especially suited for deliberating on this kind of matter. These activities were performed in line with several ethical approvals from the RUCs of the GATEKEEPER Pilots (Regional Ethics Committee CEICA—PI22/101, PI20/383, PI21/005, PI21/235—Comité de ética de la investigación con medicamentos de Euskadi (CEIm-E)—PS2021007, PS2021026, PS2021010, PS2021021, PS2020051, PS2020050—Review Bioethics Committee (RBC) which is under the Cyprus National Bioethics Committee (CNBC)—EEBK/EП/2021/25—Ethical Review Board (ERB) form by Harokopio University—Prot. Γ-2403/12.10.2020, Prot. Γ-3340/19-07-2021- Institutional Review Board of Department of Nutrition-Dietetics and then Institutional Review Board of the University of Thessaly—IRB—Prot. 2/30.11.2020, Prot. 3/07.07.2021—Institutional Review Board of UHL-Regional University Hospital of Larisa—IRB—Prot. 41210/29-09-20—Open University Human Research Ethical Committee (HREC)—HREC/3446/Antonini—HRA and Health and Care Research Wales (HCRW)—22/SC/0323—Ethical Commission of Medical University of Lodz, Poland—RNN/294/20/KE—Medical University of Lodz's Bioethical Committee—RNN/144/22/KE—Comitato etico IRCSS Istituto Tumori “Giovanni Paolo II”—Prot. N.02/CE GATEKEEPER_V1.6_03.12.2020—AReSS Ethical Committee—Verbale N. 64 23.07.2021—Ethical Committee for the Medical Faculty in Dresden—BO-EK-30012021, BO-EK-49012023).

The main aim of this was to obtain consensus through group dynamics rather than achieving statistical power, by meeting the minimum suggested threshold of 10 participants (23).

The questionnaire comprised eight sections: Consent to participate, Professional Information, and six sections concerning the actual areas of investigation, namely Engagement, Intervention, LSP Monitoring and Control, Planning, Recruitment, and Other. The questionnaire can be found in the Supplementary Data 2. The questions regarding the best practices followed a repetitive formula, namely “Please rank in order of priority the following practices, envisaged to be applied in the next large-scale Pilot, considering that resources are limited” and they were followed by an unranked list of practices which contained summary information in bold and further explanation in italics. The panellist was expected to rank the options by dragging and dropping them in the preferred order. Each area of investigation section included a box for further comments, where panellists could propose extra practices. In case new best practices were proposed by at least 30% of the respondents, they would be included in a subsequent Delphi round (18).

Results from the Delphi survey were aggregated and ranked using the Borda counting method (24). Given that the number of best practices to be ranked differs across areas, individual Borda scores were normalised at the category- and respondent-level using a min–max transformation to ensure comparability. The mean normalised score for each best practice was then derived across respondents, enabling cross-category assessment of relative ranking preferences. Higher normalised values denote practices that were more consistently placed near the top within their respective categories. To support interpretation and facilitate comparison across areas, the normalised mean Borda scores were grouped into four priority categories. This approach provides an intuitive scheme for interpreting respondents’ relative preferences for each best practice, independent of the area of investigation to which they belong. Normalised scores were assigned to priority levels using fixed thresholds: scores ≥0.25 were classified as recommended, scores ≥0.50 as highly recommended, and scores ≥0.75 as must-have. Best practices scoring below 0.25 were categorised as lower priority. These categories represent increasing levels of collective preference, ranging from practices infrequently prioritised to those consistently ranked near the top across respondents. The use of fixed thresholds ensures a transparent and easily interpretable categorisation of best practices based on their relative priority. These thresholds were established for interpretability purposes alone and should not be considered indicative of statistically significant differences between adjacent categories.

Agreement among panellists was assessed via Kendall's W test (25), using the software RStudio. For each question, Kendall's W, the associated *χ*^2^ statistic, and *p*-value were calculated to quantify the level of agreement across panellists. High values of W or of *χ*^2^, along with *p-values* lower than 0.05, were interpreted as indicative of statistically significant agreement. Based on the magnitude of Kendall's W, levels of consensus were classified as “Poor”, “Fair”, “Moderate”, “Substantial”, or “Almost Perfect”, in accordance with the categories proposed by Landis and Koch (26). Although Diamond et al. (27) report that definitions of consensus vary widely, and it is, therefore, difficult to define a “gold standard” consensus, for this study a minimum strength of agreement threshold of “Fair” was defined *a priori* as sufficient to avoid an additional Delphi round. When this threshold was not reached for a given question, a second Delphi round was conducted. This is in line with Schmidt et al. (28), who states that in Delphi studies involving a large number of items to rank, the Kendall's W value is often low.

In this round, panellists were shown the aggregated rankings derived from the first round for the non-consensus items and were asked to indicate their agreement with the proposed ranking (formulated as: “Do you agree with this ranking?”). Consensus was considered achieved if more than 50% of panellists responded positively.

**Figure 3:**
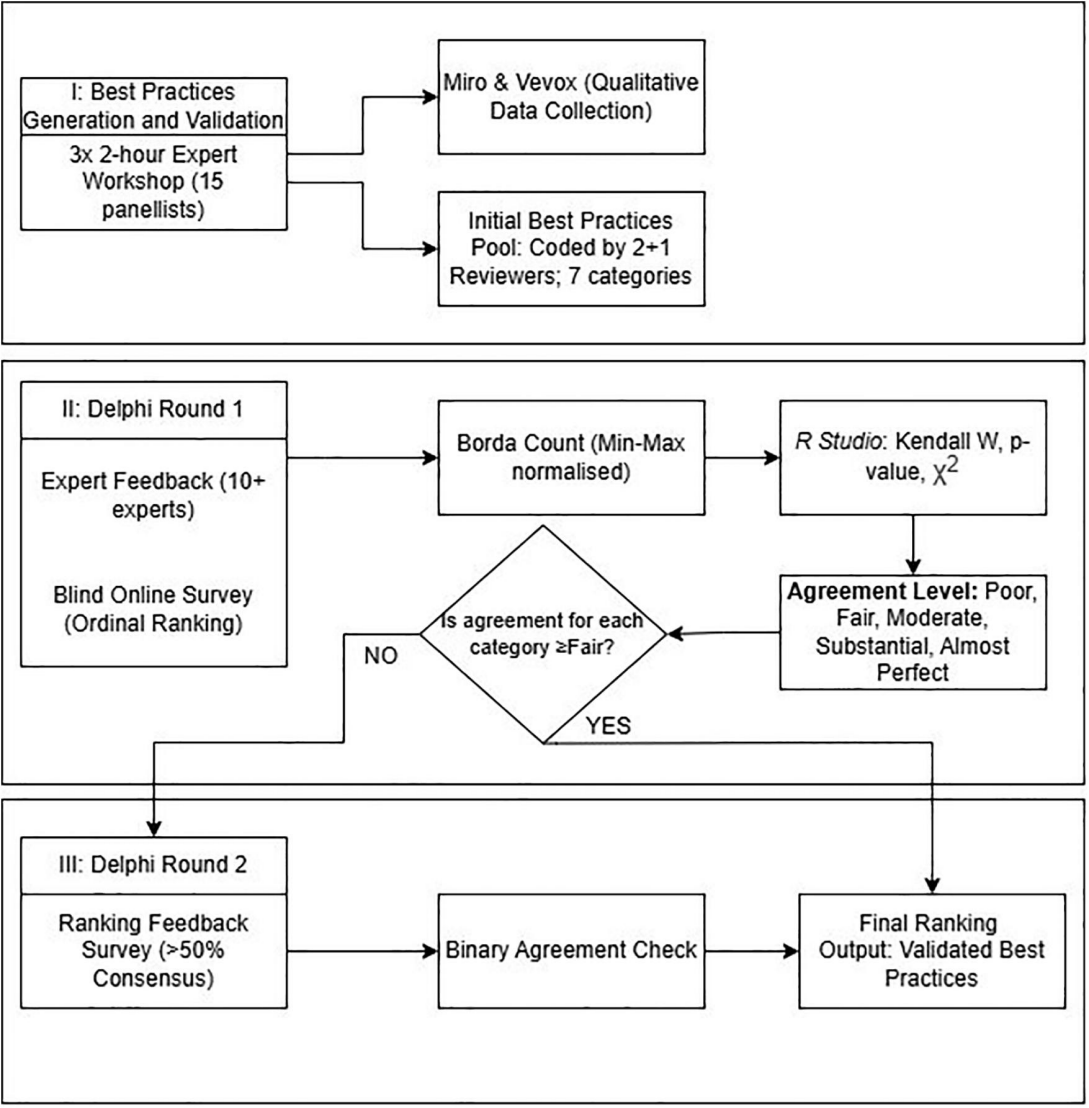
Iterative Delphi-based validation model for reaching expert consensus in LSP best practices.

The validity of the Delphi questionnaire was assessed through a pilot testing phase involving a subset of representatives distinct from the main Delphi panel. Pilot Representatives were asked to provide feedback on the clarity, relevance, and completeness of the proposed best practices, as well as to suggest additional items if necessary. Questionnaire validity was assessed to ensure that the instrument measured what it was intended to measure, following established survey evaluation principles (18). A diagram detailing each phase of the Delphi study can be found in Figure 3.

## 3 Results

### 3.1 The GATEKEEPER management framework

**Figure 4:**
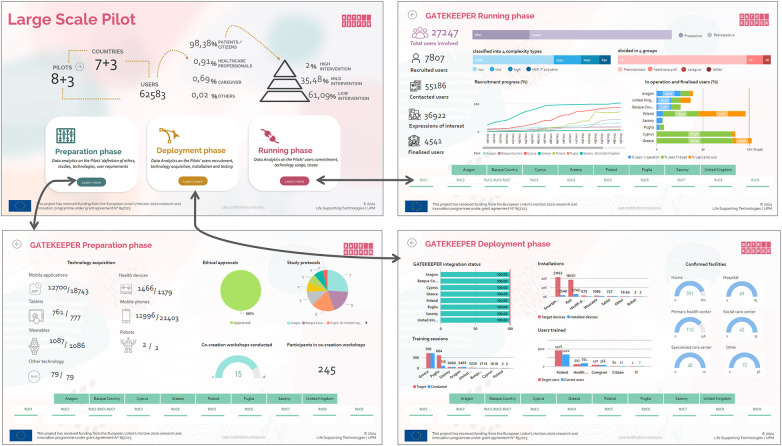
LSP progress execution MS power BI dashboard. This figure illustrates four screens from the LSP progress execution MS Power BI dashboard. The main screen provides an overview of the LSP and includes a navigation menu to access the Preparation, Deployment, and Running phase screens. Each phase screen displays relevant graphs and indicators from the measured KPIs.

The application of the Ten steps model, as described in section “Framework definition and refinement,” resulted in the GATEKEEPER management framework. The *LSP Progress execution dashboard* in MS Power BI (Figure 4), was originally conceived to feature four pages, i.e., an initial page that provides a clear overview of the tool with a menu to navigate to the other sections, and three pages, one for each phase of the Pilot — preparation, deployment and running. Nevertheless, throughout the project, the dashboard evolved to integrate new functionalities and pages. These new sections include an overview of the situation across all the Pilots in different categories, and the trajectory of the data generated throughout the project. As can be observed, this MS Power BI dashboard complements the operative KPIs reporting tool transforming data into interactive visual representations, thus intended to support the decision-making process. The *LSP Progress execution dashboard* in MS Power BI, designed for the GATEKEEPER project, can be found at this link (https://app.powerbi.com/view?r=eyJrIjoiM2U5OTliZjYtMWM1MS00YjNiLWFjMTYtMDRiM2MyODFkMTZjIiwidCI6IjZhZmVhODVkLWMzMjMtNDI3MC1iNjlkLWE0ZmIzOTI3YzI1NCIsImMiOjl9&pageName=ReportSection). The project results can be found at this link (https://cordis.europa.eu/project/id/857223).

Effective monitoring and execution of an LSP required in our case the early adoption of these tools. Within our management framework, the *Operative KPIs tool* and *LSP Progress execution dashboard* in MS Power BI tools are complemented by three additional essential components:

(A) Organisation of 2-h long monthly meetings, where each Pilot reported its status and evolution based on summary reports they submitted on an online note making tool based on the Etherpad webapp (https://etherpad.org/).
(B) Implementation log tool, consisting of a dedicated worksheet enabling Pilots to report key issues, propose mitigation actions, partners responsible for implementing such actions, and time planning (see Supplementary Data 3).
(C) *Warwick analytics tool*: based on a MS Excel spreadsheet, is a complement to the *LSP Progress execution dashboard*, providing month-by-month progress updates against pre-set targets on the ethical approval status, users recruitment, technology installation, and user training for each Pilot (see Supplementary Data 4).

Initially, this methodology was perceived to generate commitment from the Pilots and was designed to facilitate comprehensive monitoring. However, as the project progressed, the strategy proved inefficient as the intensified workload during the deployment and running phases left limited time for Pilots to provide all the requested information.

For this reason and in response to diverse and specific needs expressed by each Pilot, particularly at the RUC level, the GATEKEEPER LSP management team implemented a novel and improved, streamlined reporting and follow-up methodology, aimed at optimising the Pilots’ resources and offering enhanced support. These operational and structural changes encompass:

(A) A new short *Operative KPIs tool* reporting format that includes a minimum data set to be tracked monthly. The extended version was only filled in every 6 months.
(B) A new regular monthly meeting format, focused on the technical issues arisen since the last meeting, proactive problem-solving sessions, and official project activities preparation. This new strategy aimed to transform these meetings from unilateral reporting sessions into collaborative spaces that intended to encourage solutions co-creation.
(C) Pilots were asked to provide updates one week prior to the meeting via the above-mentioned Etherpad note making webapp, following the same initial structure, i.e., the experiment progress, the main challenges, and possible solutions.
(D) The GATEKEEPER LSP management team was tasked to analyse all the inputs provided as per A and C, and to update a table of current issues, which highlighted critical barriers and outlined necessary actions. This approach was designed to improve the efficiency of the meetings, with the goal of increasing the focus on urgent and essential points of discussion, viable solutions and next steps.
(E) At the end of the meeting, a dedicated time slot was made available for the Pilots to foster collaboration, problem-solving and to explore different alternatives to enhance their situations while exchanging experiences.
(F) The preparation of on-demand co-creation workshops to unblock issues and share best practices.

**Figure 5:**
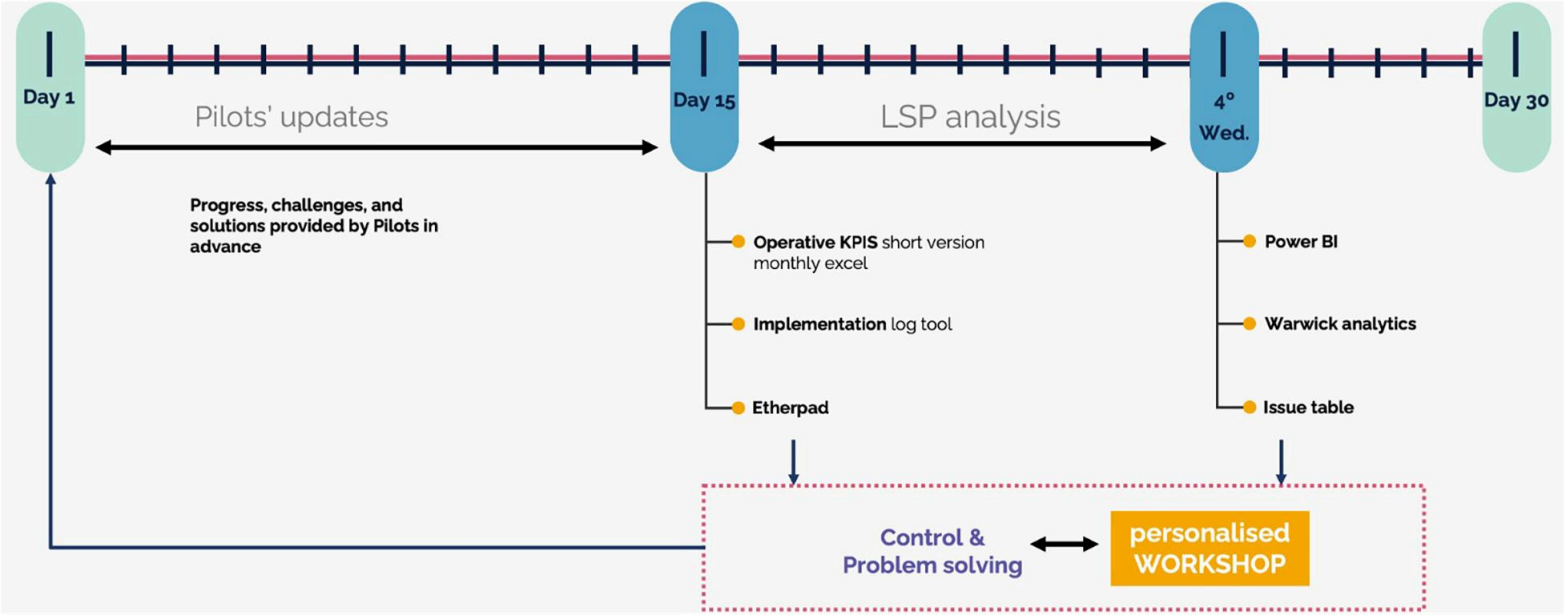
GATEKEEPER LSP monitoring framework. The diagram represents the chronological sequence of activities, tasks, tools, and users involved in the GATEKEEPER LSP monitoring framework over the course of one month.

This framework is graphically depicted in Figure 5, illustrating the whole monthly sequence of activities and tools applied. By the initial 15 days, Pilots were responsible for completing the short version of the *Operative KPIs tool* and providing progress updates, main problems and barriers encountered and potential solutions in both the Etherpad note making webapp and the Implementation log tool, illustrated above. Between the 15th of the month and the fourth Wednesday, coinciding with the monthly meeting, the GATEKEEPER LSP management team was responsible for updating the *LSP Progress execution dashboard*, the *Warwick analytics tool* and the *Issue table*. This information represented an essential element for making well-informed decisions at Pilots, internal LSP management and project level as a whole.

Future LSP project can follow a similar approach, adapting it to their specific cases. Table 1 summarises the minimum viable elements required to implement this approach and contrasts them with how they were instantiated in GATEKEEPER.

**Table 1:** Minimum viable (transferable) components vs. GATEKEEPER specific implementations.

Dimension	Minimum viable and transferable elements	GATEKEEPER specific elements
Roles	LSP Monitoring Lead (oversees KPI framework & monthly cycle) Site/Pilot Leads (provide monthly updates & execute actions) Data/BI Analyst (prepares dashboard summaries) Issue triage group (assigns and tracks mitigation actions)	LSP management team structure
Processes	Monthly monitoring loop (data → synthesis → meeting → actions) Short monthly KPI set + extended periodic dataset Standardised issue Lifecycle: capture → assign → follow up	Etherpad for pre-meeting structured inputs Specific cadence (15th of the month → synthesis → 4th Wednesday meeting) GK-specific meeting format focusing on technical blockers and co-creation
Governance	Core operative KPI set across preparation, deployment, running Versioning & refinement of KPIs/tools over time Clear responsibilities for decision & escalation pathways	LSP Management updating dashboard, analytics tool, and issue table before each meeting
Data requirements	Minimum monthly set: ethics milestones; recruitment and retention; technology readiness; engagement/training indicators; blockers & mitigation actions	Excel templates (short + extended KPI tool) Power BI dashboard pipeline Warwick analytics tool

### 3.2 Challenges in managing large-scale pilots (LSP)

The GATEKEEPER Large-Scale Pilot management framework ensured systematic oversight of Pilot progress and facilitated the early detection of implementation barriers by integrating a set of complementary tools. Through this coordinated monitoring structure, challenges were consistently identified across the preparation, deployment, and running phases, and the resulting mitigation measures were documented in a structured and transparent manner.

The most recurrent obstacle reported by Pilot sites related to delays in participant recruitment, which in several cases produced knock-on effects on technology installation, user training, and subsequent engagement activities. Recruitment difficulties often appeared together with other operational constraints, including the immaturity or instability of technological components, integration challenges with the platform, ethical and administrative delays, and limitations in staff availability. In addition, user-level barriers, particularly the low digital literacy observed among older adults, required repeated adjustments to onboarding procedures and app usability. External factors also added complexity. For example, COVID-19 temporarily reduced outpatient activity and further slowed recruitment.

Collectively, the challenges encountered and the strategies implemented to overcome them, detailed in Table 2, contributed to a continuous refinement of the GATEKEEPER management approach. Importantly, this body of experience provided the empirical foundation for the Delphi-based consensus on the best practices presented in the subsequent section, organised across the categories of Engagement, Intervention, Monitoring and Control, Planning, Recruitment, and other operational areas.

**Table 2:** Challenges in managing large-scale pilots (LSP) for eHealth deployment.

Challenge	Main consequences	Detection method	Mitigation actions
Ethics approval delays	Postponement of recruitment start Compressed operational timelines	Operative KPI admin milestones Implementation Log Monthly meetings BI dashboard	Revised/resubmitted documents Issue Table tracking Timeline adjustments Shared template examples
Recruitment delayed/targets not reached	Installations & training postponed Under-enrolment risk Reduced effective execution time	Recruitment curves Etherpad summaries Monthly minutes BI trends Warwick Analytics	Flyers, social media, expanded radius Clearer communication of benefits 1:1 recruitment (hospital, NGOs, volunteers) Workshops & stakeholder engagement Simplified onboarding & tutorials
Platform/app immaturity or instability	Deployment delays User frustration, trust decrease Data gaps	Tech KPIs Implementation Log Monthly reports BI deviations	Issue-focused meetings Targeted technical workshops Platform stabilisation & device replacement Communication of downtimes Adding help tools/tutorials
DPA/DSA bottlenecks	Delays in data flow Uncertain compliance sequence	Implementation Log (legal issues) Monthly reviews	Central tracking of blockers Coordinated resolution with partners Verification loop in Issue Table
Overburdened/fatigued HCPs	Slow recruitment Limited follow-up capacity Variability in execution	Etherpad notes KPI comparison (recruitment vs. capacity) BI workload views	Short KPI template Reduced redundancy Extra recruiters and NGO/volunteer support Peer-exchange during meetings
Low digital literacy	Difficulties in app use High demand of support Lower adherence	Support/training logs Qualitative meeting notes Implementation Log	Caregiver involvement Volunteers for installation Simplified login pages Video tutorials and in-app manuals Supportive notifications

### 3.3 Delphi study

A total of 67 people was contacted as follows: 26 key representatives from GATEKEEPER and 41 key representatives from the other projects part of OPEN DEI cluster. Additionally, they were encouraged to extend the invitation to other relevant representatives they deemed appropriate.

Our invitation got replies from 23 experts who became panellists for the first round of our Delphi study, whose details were collected and outlined in Table 3. Out of these, 58% came from the GATEKEEPER project, 31% from the PHARAON project (funded by the European Commission under grant agreement ID 857188), 4% from the HOSMARTAI project (funded by the European Commission under grant agreement ID 101016834), 4% from the VALUECARE project (funded by the European Commission under grant agreement ID 875215) and 4% did not disclose. As regards the second round of the Delphi study, 19 experts among the initial 23 replied, subdivided as follows: 70% from the GATEKEEPER project, 25% from the PHARAON project, and 5% from the HOSMARTAI project.

**Table 3:** Details of the respondents.

Respondent	Organisation	Project
1	Anonymous	ANONYMOUS
2	PASYKAF (Pagkyprios Syndesmos Karkinopathon kai Filon 1986)	GATEKEEPER
3	Biobizkaia Health Research Institute	GATEKEEPER
4	TU Dresden	GATEKEEPER
5	Carus Consilium Sachsen	GATEKEEPER
6	Multimed Engineers	GATEKEEPER
7	The Open University	GATEKEEPER
8	Technical University Dresden	GATEKEEPER
9	Servicio Aragonés de Salud	GATEKEEPER
10	Yuanpei University of Medical Technology	GATEKEEPER
11	Harokopio University	GATEKEEPER
12	Biosistemak Institute for Health system Research	GATEKEEPER
13	Fondazione Casa Sollievo della Sofferenza IRCCS	GATEKEEPER, PHARAON
14	Casa Sollievo della Sofferenza IRCCS	GATEKEEPER, PHARAON
15	Servicio Aragones de Salud	GATEKEEPER
16	INTRAS Foundation & MINDLab (ENoLL full member)	HOSMARTAI
17	Roessingh Research and Development	PHARAON
18	University of Florence	PHARAON
19	Universidad Politecnica De Cartagena	PHARAON
20	University of Florence	PHARAON
21	University of Florence	PHARAON
22	Medical University of Lodz	GATEKEEPER
23	Caritas Diocesana de Coimbra	VALUECARE and PHARAON

In both rounds, the minimum suggested threshold and common range for the size of a panel were reached (29).

The best practices are proven strategies or actions tailored to address specific categories (engagement, intervention, LSP monitoring and control, planning, recruitment, and other) in pilots, balancing agreed methods with resource constraints to enhance outcomes and pilot performance. The best practices agreed via the Delphi study are hereby reported per question and already ranked by experts, who prioritised them based on the impact of existing resource constraints. The maximum Borda score for each area is indicated in the area title in square brackets. For each best practice, both the raw Borda score and the normalised Borda score—derived using a min–max transformation—are reported in square brackets at the end of the corresponding guideline. It is important to note that a greater difference in Borda scores indicates a higher level of alignment among each expert's ranking:

- Q1: Engagement [Max. Borda score = 46]
  (A) Training sessions allow for better user engagement (lower dropouts) and overall performance. [Borda Score = 25; Normalised Borda score = 0.54]
  (B) Proactive one-to-one patients’ follow up (e.g., making sure they stick to the process) is effective in improving the Pilots performance. [Borda Score = 23; Normalised Borda score = 0.5]
  (C) Defining clear rewards/incentives and creating value for all the Pilot participants is crucial to keep them engaged. [Borda Score = 21; Normalised Borda score = 0.46]

*Resource Constraints considered*: Limited budget to provide incentives or employ personnel for one-to-one monitoring (financial and personnel constraints).

- Q2: Intervention [Max. Borda score = 115]
  (A) Digital tools should be designed to be user-friendly, readily available, and customized to meet the specific needs of end-users. [Borda Score = 95; Normalised Borda score = 0.83]
  (B) By proactively addressing digital illiteracy, the Pilot can overcome deployment hurdles and promote equal access and participation, leading to more meaningful and impactful outcomes. [Borda Score = 63; Normalised Borda score = 0.55]
  (C) Use of devices for monitoring daily activities in older adults enhances their sense of safety and ability to selfcare, thereby increasing their engagement. [Borda Score = 63; Normalised Borda score = 0.55]
  (D) Personalised messages (e-coaching) on health promotion proved to increase the adherence to the intervention. [Borda Score = 54; Normalised Borda score = 0.47]
  (E) The integration of specific digital tools in the healthcare system can facilitate their adoption. [Borda Score = 50; Normalised Borda score = 0.43]
  (F) Digital-based interventions in health, such as educational tools based on virtual reality and technology devices supporting patients, have the potential to improve early identification of adverse events, thereby increasing patient engagement. [Borda Score = 20; Normalised Borda score = 0.17]

*Resource Constraints considered*: Modern technology integration into healthcare systems entails significant costs due to the need for interoperability, compliance with regulatory requirements and, health experts training. Furthermore, bureaucratic and administrative processes often delay the start-up of interventions. Finally, there is a limited budget to employ personnel for one-to-one patient support (financial and structural constraints).

- Q3: LSP Monitoring and Control [Max. Borda score = 69]
  (A) Facilitating dialogue and information sharing among Pilots improve their performance. [Borda Score = 45; Normalised Borda score = 0.65]
  (B) Continuous monitoring of the Pilots allows for prompt intervention that could enable improvements or avoid major disruptions. [Borda Score = 40; Normalised Borda score = 0.58]
  (C) From a management point of view, minimising redundancy in the information requested from Pilots can have a positive impact on the project. [Borda Score = 38; Normalised Borda score = 0.55]
  (D) Providing digital tools to report issues and to visualise Pilots’ execution evolution and trends is helpful for them to overcome their challenges and better perform in the project. [Borda Score = 15; Normalised Borda Score = 0.22]

*Resource Constraints considered*: The personnel are limited in numbers and overburdened by the monthly pilot KPIs reporting (personnel constraints).

- Q4: Planning [Max. Borda score = 138]
  (A) Conducting usability testing prior to large-scale deployment offers numerous advantages, including improved user experience, early issue detection, enhanced solution performance, and increased adoption and retention. [Borda Score = 99; Normalised Borda score = 0.72]
  (B) The delay in the technology delivery and its lack of maturity can have a cascading effect on the motivation of Pilots involved in validating digital solutions creating uncertainty, frustration, and decreased engagement, impacting their ability to effectively test and provide valuable feedback. [Borda Score = 98; Normalised Borda score = 0.71]
  (C) Sensible and realistic planning of the intervention, its timing, and its targets is essential to sustain motivation and avoid professionals’ burnout and participants’ drop-outs. [Borda Score = 85; Normalised Borda score = 0.62]
  (D) Contingency plans dealing with the possibility of professionals’ overburden due to expected or unexpected events are needed and can potentially avoid temporary stops to the Pilot's recruitment or execution. [Borda Score = 61; Normalised Borda score = 0.44]
  (E) Defining a Pilot map/organisation chart clearly describing all actors in a given Pilot, their roles, and contact email at an early stage speeds up the communication process when an issue is detected. [Borda Score = 55; Normalised Borda score = 0.40]
  (F) Minimising the number of scheduled downtimes on the platform and informing affected users about them improves users’ confidence in the technical solution. [Borda Score = 44; Normalised Borda score = 0.32]
  (G) Easily accessible help tools such as step-by-step video tutorials and FAQs are key to facilitate participants’ registration process when it is not directly conducted by a professional. [Borda Score = 41; Normalised Borda score = 0.30]

*Resource Constraints considered*: Effective communication between stakeholders presents a significant challenge. Furthermore, difficulties in estimating the availability of technology and defining contingency plans directly impacts its delivery. Finally, the different processes and documents required by each region and country for seeking ethical approval are a major bottleneck (14) (structural and communication constraints).

- Q5: Recruitment [Max. Borda score = 138]
  (A) Active recruitment campaigns (people-based and not paper-based or online based) are more efficient than passive ones. [Borda Score = 93; Normalised Borda score = 0.67]
  (B) Personal support from health and community professionals, as well as recruitment managed by healthcare professionals, positively impact user involvement in health promotion studies by fostering trust and increasing patients’ willingness to participate. [Borda Score = 90; Normalised Borda score = 0.65]
  (C) Leveraging community initiatives (e.g., senior citizens club) improves recruitment and engagement. [Borda Score = 81; Normalised Borda score = 0.59]
  (D) Training and education of the healthcare professionals in motivating and persuading potential patients is crucial for successful recruitment. [Borda Score = 72; Normalised Borda score = 0.52]
  (E) Prioritisation of already existing and certified digital solutions instead of newly creating new technologies. [Borda Score = 71; Normalised Borda score = 0.51]
  (F) Pilots targeting a healthy population and not linked to the health system are very challenging in terms of achieving recruitment goals. [Borda Score = 55; Normalised Borda score = 0.40]
  (G) Pharmacies could be the mediator between possible users and healthcare professionals for recruitment purposes. [Borda Score = 21; Normalised Borda score = 0.15]

*Resource Constraints considered*: Limited budget to employ personnel for one-to-one patient recruitment. Additionally, healthcare professionals face time limitations, impacting their ability to participate in training programs and recruitment activities (financial and personnel constraints). Finally, recruiting and engaging healthy participants (as planned for RUC1) proved to be more challenging.

- Q6: Other [Max. Borda score = 46]
  (A) Previous experience in conducting Pilot sites brings forth numerous positive impacts on project execution and results. [Borda Score = 27; Normalised Borda score = 0.59]
  (B) Involving and identifying reference healthcare professionals from the health system organisations is essential to improve the Pilots’ performance. [Borda Score = 22; Normalised Borda score = 0.48]
  (C) Incorporating technical staff into each Pilot team improves the Pilots’ performance. [Borda Score = 20; Normalised Borda score = 0.43]

Resource Constraints considered: Not applicable.

**Figure 6:**
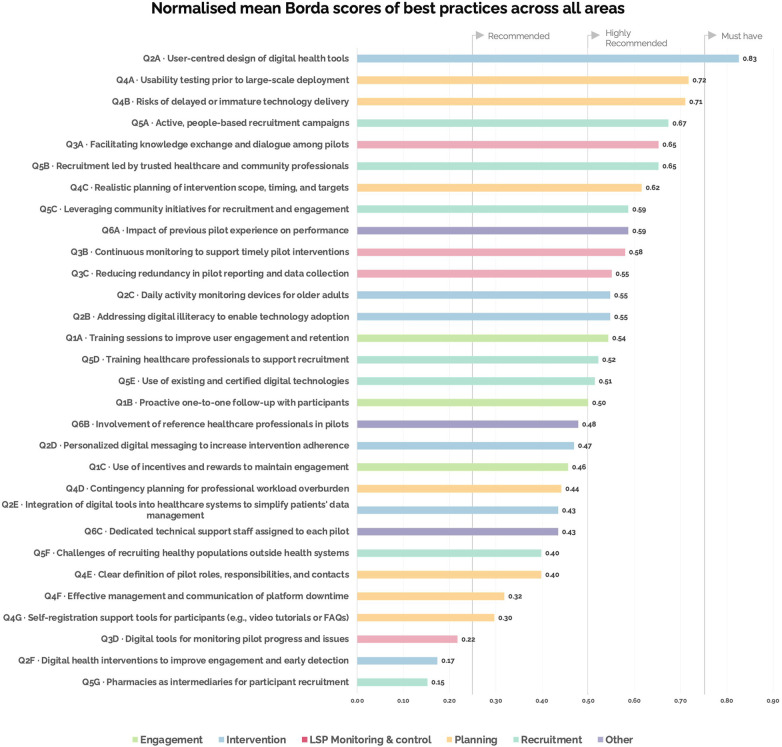
Normalised mean Borda scores of best practices across all areas. Scores range from 0 (lowest relative priority) to 1 (highest relative priority) and represent the average normalised ranking assigned by 23 respondents. Vertical reference lines indicate priority thresholds used to classify best practices into recommended (≥0.25), highly recommended (≥0.50), and must have (≥0.75) categories.

Figure 6 is a visual representation of this ranking.

As it can be observed from Table 4, only two questions (Q1 and Q6) have failed to meet the consensus requirements, with the rest of them obtaining a “Fair” level of agreement.

**Table 4:** Results and their interpretation from the statistical tests performed.

	Q1: engagement	Q2: intervention	Q3: LSP monitoring and Control	Q4: planning	Q5: recruitment	Q6: other
Kendall W	0.00756	0.318	0.202	0.248	0.248	0.0246
*χ* ^2^	0.348	36.5	13.9	34.2	34.2	1.13
*p*-value	0.84	7.4 × 10^−7^	3.04 × 10^−3^	6.28 × 10^−6^	6.28 × 10^−6^	0.568
Agreement (Yes/No)	No	Yes	Yes	Yes	Yes	No
Level of agreement	Poor	Fair	Fair	Fair	Fair	Poor

Therefore, a second round was necessary. Nineteen panellists replied to our invitation and reminder to take part in such a round. In this round, agreement was reached also in the case of Q1 and Q6. In particular, for Q1 73.7% of the panellists agreed to the proposed ranking, while for Q6 68.40% did.

## 4 Discussions and conclusions

The current manuscript presents the refined methodology applied for the management of the largest LSP project in the health domain funded by the European Commission so far, the GATEKEEPER project. The project's GATEKEEPER LSP management team developed a monitoring framework and a set of companion tools that are presented in this paper. Such tools, which were refined over time, were designed to support monitoring of the involved Pilots. These tools aimed to enable tracking of project KPIs, facilitate the collection of arising issues, and encourage co-creation. This experience allowed the GATEKEEPER LSP management team, supported by the Pilot Representatives, to collect and evaluate some key lessons learned and best practices, which were then confirmed and agreed on via a Delphi study approach.

As regards the integration of BI services, it is a key element of the proposed methodology for managing an LSP as a federation of multiple implementation studies held in multiple settings, involving thousands of participants and heterogeneous research groups. This methodology, developed and validated within the GATEKEEPER framework, employs different tools and processes for data collection, real-time monitoring, evaluation, and informed decision making, while minimising the burden on the involved stakeholders. It focuses on three main blocks, namely (1) the definition of operative KPIs essential for establishing a common framework to monitor and evaluate Pilots, regardless of their characteristics, (2) the creation of tools designed to collect results and establishment of periodic reporting, and (3) the analysis of results and trends and patterns.

Setiawan et al. (30), in their systematic review, demonstrated that operative KPIs are valuable quantitative metrics that help evaluate the success of executing activities in alignment with strategic plans. In fact, they provide insights into the LSP progress facilitating the implementation of possible contingency measures and ensuring that the project adapts effectively to unforeseen challenges. The decision to use a MS Excel spreadsheet for collecting results was primarily driven by familiarity, as most participants were already comfortable with MS Excel, online collaboration, flexibility to customise according to specific project needs, data visualisation capabilities, and seamless compatibility with tools like MS Power BI. The Operative KPIs tool template was designed to effectively track Pilot phases (preparation, deployment, and running) and to enable comparison with previous reports, serving as an independent monitoring tool. These benefits suggest that, despite the arduous task of gathering operative KPIs, this process may serve as an opportunity to optimise performance and drive informed decision-making.

In recent years, a growing trend has emerged in the investigation of how BI tools can assist healthcare systems in making more informed decisions. One critical argument presented in the study conducted by Foshay et al. (31) is that with the absence of decision-support capabilities, coupled with challenges in information accessibility, evidence-based decision-making is compromised. Additionally, there are two key barriers to effective decision-making: a lack of necessary skills and knowledge to exploit the information provided and the lack of available data. On the other hand, Kitsios et al. (32) propose a framework for the successful BI system integration in the health sector. This framework aims to improve clinical workflows and business decision-making by addressing six components: (1) involving end-users in system design, (2) streamlining data collection, processing and delivery, (3) prioritizing data quality, (4) training stakeholders, (5) establishing a clear organizational vision and (6) developing an implementation plan.

While BI tools have demonstrated their effectiveness in the assessment and decision-making process by transforming raw data into useful information presented through visual graphs and indicators in several sectors (33), there is currently a lack of evidence on their impact on eHealth LSP monitoring. Foshay and Kuziemsky highlight in their study regarding the implications of integrating BI tools into the hospital system (31), that accurate and timely information is essential for making evidence-based decisions that can be trusted. Consequently, BI tools are conceived as an opportunity to reduce risks and accelerate decision-making processes during the execution of an LSP. Specifically, within the GATEKEEPER context, the *LSP Progress execution dashboard* in MS Power BI was perceived by the team to have contributed positively. Throughout the project, it evolved to integrate not only new metrics for monitoring Pilot execution but also other Pilot related activities, such as AI models’ development. This dashboard provides clear insights into the overall LSP status and individual Pilots’ performance over time. Consequently, integrating it into monthly follow-up meetings may facilitate trends identification, improve communication, and accelerate decision-making. Following several iterations and refinements, these tools have reached a mature version that appeared to be well-accepted by both the Pilots and the GATEKEEPER LSP management team, based on informal feedback. Via these tools, the former have to periodically report the results obtained, while the latter is in charge of the design, implementation, and transfer of the data to the *LSP Progress execution dashboard* in MS Power BI for subsequent analysis. It is important to highlight that the complete system has been designed with an adaptable and scalable structure, facilitating integration in future LSPs.

As illustrated, the approach outlined in this manuscript aligns with these insights guaranteeing: (1) a robust set of Operative KPIs for efficient monitoring of LSP execution, (2) an adaptable template for Operative KPIs collection that safeguards data quality and integrity, (3) an intuitive and user-centric dashboard to empower decision-making and the identification of trends and risks, and a log tool for (4) gathering contingency measures to mitigate potential barriers and reduce risks.

Overall, the use of a BI-supported management process was perceived to significantly enhance decision-making, efficiency, and resource allocation by providing immediate insights into the current status and trends of the project through advanced data visualisation and analytics. This enabled stakeholders to quickly identify issues, track progress, spot patterns, and predict future outcomes, facilitating informed decisions based on up-to-date information. Unlike traditional small-scale pilots, which typically involve a single use case and a common setting across participating centres, our approach required standardising the visualisation and analysis of diverse pilots in various settings, each with unique goals in terms of recruitment, interventions, follow-up, and outcomes. By establishing a common benchmark for pilot performance, we minimised the burden on the pilots while ensuring comprehensive and consistent monitoring.

As regards our consensus-based lessons learned, they were presented in the form of ranked best practices, divided into six categories, namely Engagement, Intervention, LSP monitoring and control, Planning, Recruitment, Other. These areas reflected the challenges that we experienced during the project. Notably, the resulting ranking reflects a balance between the existing resource constraints associated with implementing these best practices and their effectiveness.

Starting from the Engagement, it was one of the great challenges encountered. In fact, keeping participants engaged with the intervention, especially if they were not patients but ordinary citizens (RUC1 intervention) turned out to be quite difficult. Nkyekyer et al. (34) reported several studies that, although successful in the recruitment phase, faced substantial issues related to engagement. Similarly to their study, in this paper, incentivising rewards and providing 1–1 follow up were identified by experts as potentially effective solutions. Nonetheless, online training sessions are perceived as the most influential engagement strategy, receiving the highest normalised Borda score among all practices in this category. This highlights that structured, accessible, and well-designed training remains essential for ensuring users understand the intervention, feel confident using the tools, and are less likely to disengage over time (35).

In terms of the Intervention area, user-friendly, readily available, and tailored digital tools constitute the most critical determinant of successful intervention uptake, receiving by far the highest normalised Borda score among all Intervention practices. This strong consensus reinforces that digital health solutions must minimise usability barriers and be adapted to the needs and capabilities of diverse user groups to support effective large-scale deployment. Similarly, but less impactfully, prioritising the addressing of digital illiteracy plays a central role in enabling equitable participation and overcoming deployment challenges, particularly in older or vulnerable populations. Equally, the experts also attributed moderate importance to monitoring devices that enhance users’ sense of safety and self-care, recognising their potential to strengthen engagement by providing reassurance and real-time feedback, as also reported by Alotaibi et al. (35).

As regards the Monitoring and Control area, relying on efficient ad-hoc monitoring tools, limiting the number of requests to Pilots to a manageable number, and fostering co-creation and dialogue, were seen as paramount. This is in line with evidence found in literature (36–38).

In terms of the Planning category, conducting usability testing prior to large-scale deployment emerged as the highest-priority practice, achieving the strongest normalised Borda score. This confirms that early assessment of usability is essential not only for improving user experience but also for identifying functional issues and mitigating adoption barriers before scaling (39, 40). Closely following in the ranking, experts emphasised the substantial impact of technology delivery delays and immaturity, recognising that unstable or late-stage technologies can undermine professionals’ motivation, create uncertainty, and compromise the ability to provide meaningful feedback during Pilots. The prioritisation of realistic planning of interventions, timelines, and targets further aligns with our experience, as well-structured plans were crucial for maintaining stakeholder engagement and preventing operational burnout during GATEKEEPER's execution, as also reported by (38, 40).

As regards the Recruitment area, it was noticed that the recruitment for studies targeting healthy populations presents significant challenges, as also reported by Coday et al. (41). Nonetheless, independent of the target population, our ranking shows that the top best practice in this category is active and people-based recruitment campaigns. This reflects the continued importance of personal interaction, trust, and direct communication in motivating individuals to participate, particularly in complex or unfamiliar digital health interventions. Closely following in priority, experts attributed relevant influence to recruitment driven by trusted healthcare and community professionals, recognising that their involvement fosters credibility and increases participants’ willingness to join studies. Ramachandran et al. stress the same concepts as these two best practices (42). The ranking also highlights the value of leveraging existing community initiatives, such as senior clubs or local associations, which can provide well-established social structures that facilitate smoother recruitment and sustained engagement, as also reported by George et al. (43). Furthermore, training healthcare professionals in persuasive communication was identified as an important contributor to recruitment success, confirming that the skills and confidence of recruiters play a significant role in shaping participant decisions. Interestingly, the ranking places prioritisation of already established digital solutions moderately high, suggesting that relying on mature and certified technologies may reduce hesitancy and improve adoption during the recruitment phase. As also reflected in GATEKEEPER's own experience, experts acknowledged that recruiting healthy individuals not linked to healthcare pathways presents particular challenges, receiving a mid-ranking position due to the additional motivation and outreach efforts required. Finally, while pharmacies as recruitment mediators ranked lowest, their role was not dismissed; rather, experts viewed them as a complementary channel with potential value in specific contexts. Overall, the revised ranking suggests that recruitment strategies for LSPs must remain inherently people-centred, leveraging trusted relationships, established community infrastructures, and skilled communication to overcome participation barriers.

Finally, as regards the *Other* area, previous experience in conducting Pilot sites was regarded as the most effective best practice, followed by involving and identifying reference healthcare professionals from the health systems organisation. This is related to their ability to solve complex problems and to bring forward valuable insights to practical challenges that may arise (44).

### 4.1 Strengths and limitations

Overall, in evaluating this study, it is essential to consider both its strengths and limitations. The study features several points of strength, including the diversity of settings, encompassing various regions, health systems, and target populations with different disease severities and needs, all managed under a common system, i.e., a federation of multicentre longitudinal cohort studies. This diversity may support the applicability of the results across different contexts, although this may require further empirical validation. Additionally, the study's size (over 27,000 involved users) and duration (51 months) enabled the development, testing, and refinement of KPIs and tools, as well as the opportunity to observe the evolution of management approaches over several years, though direct impact assessment was not systematically measured. The use of co-creation approaches further enhances the study by involving relevant stakeholders in the development process, ensuring that the solutions are practical and tailored to real-world needs. Moreover, the best practices have been confirmed and agreed on through a Delphi approach, ensuring the reliability and consensus of the findings. Borda counting, the selected voting and consensus scheme for the Delphi study, is one of the most reliable and simple techniques for this purpose (45).

Despite its contributions, several limitations warrant consideration. First, the study's internal validity is constrained by a heavy reliance on the GATEKEEPER project for framework development and validation. Although the Delphi study incorporated experts from four EU-funded Large-Scale Pilots (LSPs), the significant concentration of panellists from the GATEKEEPER project (60%–70% across rounds) and a geographic skew toward Southern and Western Europe may have introduced institutional and regional biases. This concentration potentially limits the transferability of our findings to LSPs with different governance structures or regulatory environments. For this reason, we framed this from the point of view of “multi-project expert consensus building” rather than “external validation”. Second, the reliance on Borda counting presented challenges, as panellists found it difficult to rank numerous options within single categories—a friction reflected in low consensus indicators such as Kendall's W. In future studies, it is recommended to rely on Likert-scale type scales rather than pure option ranking to reduce the impact of this operational heterogeneity. Furthermore, the study lacks empirical evidence of causal relationships between specific management interventions and KPI improvements. Given the complexity of LSP ecosystems, establishing direct causality remains difficult and would require controlled comparisons beyond the current scope. While this framework has demonstrated high operational utility across the GATEKEEPER Pilots, further research would be required to quantitatively measure its long-term impact on projects.

Finally, our dependence on self-reported feedback and internal documentation may have introduced social desirability bias. While these best practices represent a multi-project consensus based on expert experience, future research should employ independent external evaluations and broader empirical testing across diverse LSP contexts to strengthen the framework's external validity.

In conclusion, the size and scope of LSP projects makes it necessary to plan for appropriate projects’ monitoring and surveillance tools. This paper presented our *modus operandi* in the management of the GATEKEEPER LSP project, one of the largest trailblazing projects of AI applied to healthcare in Europe. This project is well situated in the current evolving digital health research panorama globally, where a shift towards Healthcare 4.0 and 5.0 is taking place. Managing and supporting such a large LSP project comes with several challenges. In this paper we aimed to look back at the main challenges and how these have affected and improved the way we approached project management, distilling all we learned into effective and useful best practices that were confirmed and agreed to by representatives of other recent LSP projects in Europe via a Delphi study approach. Such best practices may inform the planning, management, and delivery of future LSP projects, potentially contributing to their success.

## Data Availability

The original contributions presented in the study are included in the article/Supplementary Material, further inquiries can be directed to the corresponding author.

## The GATEKEEPER Consortium

Applied Biomedical Signal Processing Intelligent eHealth Lab, University of Warwick, CV47AL, Coventry, United Kingdom: Silvio M. Pagliara. Group of Translational Research in Respiratory Medicine, IRBLleida, Hospital Universitari Arnau de Vilanova and Santa Maria, Centro de Investigación Biomédica en Red de Enfermedades Respiratorias (CIBERES), Lleida, Spain: Ferran Barbé and Gerard Torres. Life Supporting Technologies-LifeSTech, Universidad Politécnica de Madrid, 28040, Madrid, Spain: Maria Fernanda Cabrera, Laura Lopez, and Maria Teresa Arredondo. Internationalization and Project Coordination Unit, Biobizkaia Health Research Institute, Plaza de Cruces 12, 48903, Barakaldo (Bizkaia), Spain: Eunate Arana Arri and Janire Orcajo. Biosistemak Institute for Health System Research, Bizkaia, Spain; Network for Research on Chronicity, Primary Care, and Health Promotion (RICAPPS), Spain: ne Fullaondo. Department of Family Medicine, Medical University of Lodz, Łódź, Poland: Piotr Klimczak and Ewelina Łojewska. Faculty of Medicine Carl Gustav Carus, Technical University Dresden, Germany: Kai Gand. Puglia Pilot: Francesco Ricciardi and Stella Pastore. Medtronic Ibérica, C. de María de Portugal, 11, Hortaleza, 28050 Madrid, Spain: Paula Curras, Jorge Posada, German Gutierrez. MYSPHERA, Ronda Auguste y Louis Lumière, 23, Nave 13, 46980 Paterna, Valencia, Spain: Sergio Guillen. Department of Nutrition and Dietetics, School of Health Science and Education, Harokopio University, Athens, Greece: Yannis Manios and Eva Karaglani. University of Thessaly/Digital Cities of Central Greece: Odysseas Androutsos, Maria Vlachava, Alexandra Bargiota and George E. Dafoulas. Oncology Department, Ysbyty Gwynedd, Betsi Cadwaladr University Health Board, Bangor, United Kingdom; Warwick Medical School & Cancer Research Centre, University of Warwick, Coventry, United Kingdom; Chronotherapy, Cancers and Transplantation Research Unit, Faculty of Medicine, Université Paris-Saclay, Villejuif, France: Pasquale Innominato. Department of Medical Sciences, Fondazione IRCCS Casa Sollievo Della Sofferenza, San Giovanni Rotondo, Foggia, Italy: Salvatore De Cosmo, Antonio Mangiacotti. Fondazione Politecnico di Milano, Milano, Italy: Paolo Paolini. Innovation and Procurement Officer, InnovaPuglia S.p.A., https://www.innova.puglia.it/en_GB: Marco di Ciano. EU initiatives Project Manager, InnovaPuglia S.p.A., https://www.innova.puglia.it/en_GB: Antonio Campese. Managing Director, Agenzia Regionale per la Salute ed il Sociale (AreSS) Puglia, https://aress.regione.puglia.it/en/web/guest: Francesco Fera.
