# Supplementary material for: Best practices and challenges in executing large scale pilots for eHealth deployment and managing innovation: the GATEKEEPER experience
Source: Front Digit Health. 2026 May 29;8:1730232. doi: 10.3389/fdgth.2026.1730232 (PMC13260382; doi:10.3389/fdgth.2026.1730232)
Supplement: Supplementary file 1 [file Supplementaryfile1.pdf]

This project has received funding from the European Union's Horizon 2020 research and innovation programme under grant agreement Nº 857223

Lessons learned for large scale pilot management and digital health deployment in EU

The following Delphi study (10 min) has been elaborated by the GATEKEEPER project, which is a Horizon 2020 research project funded by the European Commission and conducted by an international team of researchers. With this survey, we aim to gather your feedback on the impressions that the large scale pilot (LSP) management team from the Gatekeeper project collected. These impressions relate to the best practices and lessons learned regarding the management of LSPs and the deployment of digital health on a large scale in Europe.
If you agree to join, you will be one of the panelists of this study. We aim to prioritise such impressions based on your answers as well as measure your level of consensus. Thank you for taking your time to complete this.

* Obbligatoria

CONSENT TO PARTICIPATE

1. Do you agree to take part to this study and become a panelist? *

◯ Yes

◯ No

PROFESSIONAL INFORMATION

2. Institution *

3. LSP Project (e.g., Gatekeeper) *

4. Pilot and RUC (e.g., Basque Country, RUC 7) *

5. Email (this is just needed to include you in a second round of the Delphi study, if needed) *

ENGAGEMENT

6. Please rank in order of priority the following practices in terms of engagement envisaged to be applied in the next large scale pilot, considering that resources are limited.

   Please notice that if you agree with the initial ranking (i.e., the one you are presented with when first seeing the question) you will need to modify it and go back to the original one, or Microsoft Forms won't let you go forward. *

   - **ENGAGEMENT: Proactive one-to-one patients' follow up (e.g., making sure they stick to the process) is effective in improving the Pilots performance.** *Further explanation: Contacting end-users individually, guiding them to ensure they are completing the envisaged tasks and giving them feedback has a positive impact on pilots' performance.*
   - **ENGAGEMENT: Defining clear rewards/incentives and creating value for all the pilot participants is crucial to keep them engaged.** *Further explanation: Participants are more engaged with the solution if they receive rewards or incentives and if the benefit obtained from taking part in the intervention is clear. Examples of these incentives are gift cards, vouchers or include gamification elements in the solution (points, ranking, etc).*
   - **ENGAGEMENT: Training sessions allow for better user engagement (lower dropouts) and overall performance.** *Further explanation: Pilots undergoing training sessions will benefit from this in terms of user engagement as they know better the technology they have to use and their benefits.*

7. Please use this box to suggest further impression related to this section that you may have (optional).

INTERVENTION

8. Please rank in order of priority the following practices in terms of intervention envisaged to be applied in the next large scale pilot, considering that resources are limited.

   Please notice that if you agree with the initial ranking (i.e., the one you are presented with when first seeing the question) you will need to modify it and go back to the original one, or Microsoft Forms won't let you go forward.
   *

- **INTERVENTION: Digital-based interventions in health, such as educational tools based on virtual reality and technology devices supporting patients, have the potential to improve early identification of adverse events, thereby increasing patient engagement.** *Further explanation: By leveraging technological tools and real-time health data, these interventions enable timely interventions, empower patients to take proactive measures, and enhance overall patient engagement in their healthcare journey.*

- **INTERVENTION: The integration of the specific digital tools in the healthcare system can facilitate their adoption.** *Further explanation: Full integration of digital tools into the local healthcare system to simplify patients' data management can facilitate their adoption if their implementation doesn't require significant extra effort.*

- **INTERVENTION: By proactively addressing digital illiteracy, the pilot can overcome deployment hurdles and promote equal access and participation, leading to more meaningful and impactful outcomes.** *Further explanation: If people are not familiar with how to use technology, it is more difficult to run successfully a pilot that implies the use of technology without previous training.*

- **INTERVENTION: Personalized messages (e-coaching) on health promotion proved to increase the adherence to the intervention.** *Further explanation: Receiving personalized messages based on age, pathologies or preferences that promote a healthy lifestyle increases the adherence to the intervention as they only receive information that is of the users' interest.*

- **INTERVENTION: Digital tools should be designed to be user-friendly, readily available, and customized to meet the specific needs of end-users.** *Further explanation: Usability guidelines and specific end-user needs should be taken into account when a digital tool is developed. Digital tools like mobile or web applications should guide users and explain the purpose of functionalities, provide error feedback, and incorporate shortcuts to streamline navigation, etc.*

- **INTERVENTION: Use of devices for monitoring daily activities in older adults enhances their sense of safety and ability to selfcare, thereby increasing their engagement.** *Further explanation: Incorporating into daily life tools that help monitor parameters such as steps, distance, minutes of activity, sleep, blood pressure, heart rate, temperature, etc. and using these parameters for sending notifications improves the sense of safety and thereby the trust in these solutions and their daily use.*

9. Please use this box to suggest further impression related to this section that you may have (optional).

LSP MONITORING AND CONTROL

10. Please rank in order of priority the following practices in terms of LSP monitoring and control, envisaged to be applied in the next large scale pilot, considering that resources are limited.

    Please notice that if you agree with the initial ranking (i.e., the one you are presented with when first seeing the question) you will need to modify it and go back to the original one, or Microsoft Forms won't let you go forward.
    *

- **LSP MONITORING AND CONTROL: Facilitating dialogue and information sharing among Pilots improve their performance.** *Further explanation: Offering a discussion space where pilots can share information about the main issues and challenges and how they have overcome them is valuable information for other pilots that are in the same situation.*
- **LSP MONITORING AND CONTROL: Providing digital tools to report issues and to visualize pilots' execution evolution and trends is helpful for them to overcome their challenges and better perform in the project.** *Further information: Pilots can use different digital tools offered by the management team: (1) business analytics tool to study the trends and the areas that should be improved (this tool includes charts, filters, indicators, statistics and the evolution of the pilots' execution based on the KPIs provided by pilots), (2) collaborative reporting tool to report issues and possible mitigation actions and (3) communication app to interact with technical team to solve technical issues.*
- **LSP MONITORING AND CONTROL: From a management point of view, minimizing redundancy in the information requested from pilots can have a positive impact on the project.** *Further information: It increases efficiency, improves pilot engagement and satisfaction, enhances data accuracy and reliability, promotes clearer communication channels, fosters collaboration and trust, and leads to time and cost savings.*
- **LSP MONITORING AND CONTROL: Continuous monitoring of the Pilots allows for prompt intervention that could enable improvements or avoid major disruptions.** *Further information: It is likely that Pilots' intermediate results can guide a better design of the intervention Action Plan to achieve the expected results. For instance, KPIs like contacted users and interested users could be useful to explore this.*

11. Please use this box to suggest further impression related to this section that you may have (optional).

PLANNING

12. Please rank in order of priority the following practices in terms of Planning, envisaged to be applied in the next large scale pilot, considering that resources are limited.

    Please notice that if you agree with the initial ranking (i.e., the one you are presented with when first seeing the question) you will need to modify it and go back to the original one, or Microsoft Forms won't let you go forward.
    *

- **PLANNING: Conducting usability testing prior to large-scale deployment offers numerous advantages, including improved user experience, early issue detection, enhanced solution performance, and increased adoption and retention.** *Further information: Testing the app with real users allows for the identification of any functional or design flaws early in the development process while developers gain valuable feedback on users' preferences, expectations, and pain points.*
- **PLANNING: Contingency plans dealing with the possibility of professionals' overburden due to expected or unexpected events are needed and can potentially avoid temporary stops to the pilot's recruitment or execution.** *Further information: Healthcare professionals are exposed to both expected and unexpected work peaks, such as seasonal viral infections or health emergencies, that may overburden them and limit their commitment to a research project. Therefore, it is key to have contingency plans clearly stating what should be done in such scenarios and who must do what.*
- **PLANNING: Minimizing the number of scheduled downtime on the platform and informing affected users about them, improves users' confidence in the technical solution.** *Further explanation: Appropriately dealing with platform downtimes, ensuring correct and timely advance notification to both professionals and participants helps in building trust towards the platform.*
- **PLANNING: Easily accessible help tools such as step by step video tutorials and FAQs are key to facilitate participants' registration process when it is not directly conducted by a professional.** *Further information: Having participants registering on their own (and some times even downloading and installing required Apps) may be very challenging for older participants. Therefore, development of easy step-by-step tutorials and other helpful content may be crucial to overcome barriers in the self-registration process. Moreover, the existence of such tools (if done professionally) already helps in building trust in the project/pilot/solution.*
- **PLANNING: Defining a pilot map/organization chart clearly describing all actors ina given pilot, their roles and contact email at an early stage, speeds up the communication process when an issue is detected.** *Further explanation: Creation of such an organization chart is helpful not only to identify missing actors or untackled tasks, but also to ease communication and efficiently tackle problems/challenges.*
- **PLANNING: The delay in the technology delivery and its lack of maturity can have a cascading effect on the motivation of pilots involved in validating digital solutions creating uncertainty, frustration, and decreased engagement, impacting their ability to effectively test and provide valuable feedback.** *Further information: Technology delivery frequently emerges as a significant bottleneck with a profoundly adverse effect on the successful implementation of pilots.*
- **PLANNING: Sensible and realistic planning of the intervention, its timing, and its targets is essential to sustain motivation and avoid professionals' burnout and participants' drop-outs.** *Further information: Professionals and participants need to know what is expected from them from the very beginning. Moreover, it is crucial that what is required from them is feasible and can realistically be done without implying too much effort.*

13. Please use this box to suggest further impression related to this section that you may have (optional).

RECRUITMENT

14. Please rank in order of priority the following practices in terms of Recruitment, envisaged to be applied in the next large scale pilot, considering that resources are limited.

    Please notice that if you agree with the initial ranking (i.e., the one you are presented with when first seeing the question) you will need to modify it and go back to the original one, or Microsoft Forms won't let you go forward.
    *

- **RECRUITMENT: Personal support from health and community professionals, as well as recruitment managed by healthcare professionals, positively impact user involvement in health promotion studies by fostering trust and increasing patients' willingness to participate.** *Further explanation: Healthcare professionals are usually seen as highly trustworthy by their patients thus making these professionals highly suited for informing and recruiting participants.*
- **RECRUITMENT: Prioritization of already existing and certified digital solutions instead of newly creating new technologies.** *Further explanation: Using already stablished technologies simplifies implementation, and avoids participants/professionals burnout caused by the use of partially developed tools.*
- **RECRUITMENT: Training and education of the healthcare professionals in motivating and persuading potential patients is crucial for successful recruitment.** *Further explanation: Healthcare professionals' proficiency in the technologies used in interventions is not always optimal, but providing them with adequate training can significantly enhance their confidence, sense of security, and engagement with end-users.*
- **RECRUITMENT: Leveraging community initiatives (e.g., senior citizens club) improves recruitment and engagement.** *Further explanation: When aiming to recruit participants not directly contacting the health system, it is key to do so in trusted environments frequented by the target population. This includes, for instance, senior citizen clubs,social premises of the town hall, etc.*
- **RECRUITMENT: Active recruitment campaigns (people-based and not paper-based or online based) are more efficient than passive ones.** *Further explanation: It is likely that people are more likely to be involved in the study if guided by active recruitment campaigns.*
- **RECRUITMENT: Pilots targeting healthy population and not linked to the health system, are very challenging in terms of achieving recruitment goals.** *Further explanation: Participants' willingness to participate in a study is usually linked to a balance of their self-foreseen participation benefits and costs (mostly in terms of time to answer questionnaires, use of medial devices, etc). Therefore, it is easier to recruit participants that are patients in a healthcare system, as they are in need of care and are willing to receive additional attention, and enhanced care. In contrast, healthy participants value their time more than their health or their contribution to science.*
- **RECRUITMENT: Pharmacies could be the mediator between possible users and healthcare professionals for recruitment purposes.** *Further explanation: Identification of intermediate recruiting/contact agents such as personnel in pharmacies can boost recruitment and sustain participants'engagement.*

15. Please use this box to suggest further impression related to this section that you may have (optional).

OTHER

16. Please rank in order of priority the following practices, envisaged to be applied in the next large scale pilot, considering that resources are limited.

    Please notice that if you agree with the initial ranking (i.e., the one you are presented with when first seeing the question) you will need to modify it and go back to the original one, or Microsoft Forms won't let you go forward.
    *

- **OTHER: Involving and identifying reference healthcare professionals from the health system organizations is essential to improve the pilot performance.** *Further explanation: Involving healthcare professionals the pilot can benefit from their expertise and experience to improve outcomes and effectiveness and helps ensure that the project is aligned with the best practices and standards established in the healthcare system.*
- **OTHER: Incorporating technical staff into each pilot team improves the Pilots' performance**. *Further explanation: In a Large Scale Pilot project context with different interventions, given the heterogeneity of the solutions, is more effective to have a dedicated technical representative per pilot to fix possible technical issues instead of depending on a common technical representative for all the pilots.*
- **OTHER: Previous experience in conducting pilot sites brings forth numerous positive impacts on project execution and results.** *Further explanation: Pilots with previous experience in the domain will perform better: enhanced planning and execution, improved methodologies and documentation, efficient issue identification and resolution, enhanced stakeholder engagement, and streamlined knowledLeveraging this experience sets the stage for a more successful pilot phase and contributes to the overall success of the project.*

17. Please use this box to suggest further impression related to this section that you may have (optional).

18. Please use this box to add any further comment (optional)

Questo contenuto non è stato creato né approvato da Microsoft. I dati che invii verranno recapitati al proprietario del modulo.

Microsoft Forms
